# Multivariate analysis of factors associated with kyphotic deformity after laminoplasty in cervical spondylotic myelopathy patients without preoperative kyphotic alignment

**DOI:** 10.1038/srep43443

**Published:** 2017-02-27

**Authors:** JunMing Cao, JingTao Zhang, DaLong Yang, Liu Yang, Yong Shen

**Affiliations:** 1 Department of Orthopedics, The Third Hospital of Hebei Medical University, Shijiazhuang, Hebei, People’s Republic of China; 2 Department of Spinal Surgery, The Third Hospital of Hebei Medical University, Shijiazhuang, Hebei, People’s Republic of China

## Abstract

The risk factors of post-laminoplasty kyphosis in patients with cervical spondylotic myelopathy (CSM) without preoperative kyphotic alignment are not well known. This study aimed to compare clinical and radiological data between patients with or without post-laminoplasty kyphosis and to investigate the factors associated with post-laminoplasty kyphosis in CSM patients without preoperative kyphotic alignment. Patients (n = 194) who received unilateral expansive open-door cervical laminoplasty with miniplate fixation and completed a 1-year follow-up were enrolled. Patients were grouped according to whether they suffered from postoperative kyphosis (P) or not (NP). Postoperative kyphosis was observed in 21 (10.8%) patients. The recovery rates of the Japanese Orthopaedic Association scores at the 1-year follow-up in the P group were inferior to those in the NP group (31.9% vs. 65.2%, P < 0.001). Logistic regression with post-laminoplasty kyphosis as the dependent variable showed independent risks associated with an increased C2–7 sagittal vertical axis (SVA, odds ratio [OR] = 1.085, 95% confidence interval [CI] = 1.025–1.203, P = 0.015), destroyed facet joints (OR = 1.132, 95% CI = 1.068–1.208, P < 0.001), and cephalad vertebral level undergoing laminoplasty (CVLL, OR = 2.860, 95% CI = 1.164–6.847, P = 0.021). These findings suggest that CVLL, C2–7 SVA, and destroyed facet joints are associated with kyphosis after laminoplasty in CSM patients without preoperative kyphotic alignment.

Unilateral expansive open-door cervical laminoplasty is widely used for treating patients with cervical spondylotic myelopathy (CSM)^1^,^2^,^3^. Adequate decompression can be obtained when cervical lordosis is maintained to allow a posterior shift of the spinal cord after laminoplasty. Although preoperative cervical alignment is normal, kyphotic deformity can occur after cervical laminoplasty^4^. Baba *et al*.^5^ showed that cervical lordosis following laminoplasty is associated with posterior migration of the cervical spinal cord. They also showed that posterior cord migration correlates with improved outcomes based on the Japanese Orthopaedic Association (JOA) score. Therefore, post-laminoplasty kyphotic deformity could greatly affect neurological function. The factors leading to post-laminoplasty kyphotic deformity are complex. Several possible factors have been proposed, including age, preoperative cervical sagittal malalignment, destruction of posterior structures, posterior muscle dystrophy, and the cephalad vertebral level undergoing laminoplasty (CVLL)^6^,^7^,^8^. However, the mechanisms of post-laminoplasty kyphotic deformity have not been fully clarified yet.

The current study aimed to compare clinical and radiological data between patients with or without post-laminoplasty kyphotic deformity. We also aimed to determine the potential factors associated with post-laminoplasty kyphotic deformity by multivariate analysis in patients with CSM without preoperative kyphotic alignment.

## Methods

### Ethics statement

The study was approved by the Ethics Committee of the Third Hospital of Hebei Medical University in China. There was no need to obtain informed consent from patients because this was a retrospective study and all of the data were collected and analysed anonymously. The methods were carried out in accordance with the approved guidelines.

### Patient population

This retrospective study included 194 consecutive patients who underwent unilateral expansive open-door cervical laminoplasty for CSM in the Third Hospital of Hebei Medical University between January 2010 and July 2015. Exclusion criteria were as follows: cervical ossification of the posterior longitudinal ligament, cervical disc herniation, a preoperative C2–7 lordotic angle <−5 degrees, a previous history of cervical spine surgery, combined congenital abnormality, and patients who could not maintain an upright position without assistance. Preoperatively, all of the patients had lordotic or straight sagittal alignment (C2–7 lordotic angle >−5 degrees) because we generally performed anterior decompressive surgery or posterior decompression with fusion for such patients with preoperative kyphotic alignment at our institution. All of the patients were followed for at least 12 months.

### Operative procedure

The location and number of levels that were treated surgically were considered based on magnetic resonance imaging (MRI) or computed tomography (CT). If the cephalad extent of spinal stenosis was no further cephalad than the C3–4 intervertebral level, laminoplasty starting at the C4 level was performed. For patients who had spinal cord compression at the C2–3 level, the inferior lamina at C2 was fenestrated, and laminoplasty starting at the C3 level was performed. The side with more symptoms was used as the opening side. A drill with a matchstick bur was used to open the hemilamina on the side that was associated with more symptoms. A shallow trough was created on the contralateral hemilamina with the same drill bit, and this side was used as a hinge to open the laminoplasty. Open-door laminoplasty was secured using an appropriately-sized titanium miniplate (Centerpiece Plate Fixation System; Medtronic Sofamor Danek, USA). Small screws were then placed through the plate apertures into the lateral mass on one side and into the opened hemilamina on the other side. All of the patients were encouraged to perform early neck exercises until 2 weeks after the surgeries.

### Outcome measures

**Figure 1:**
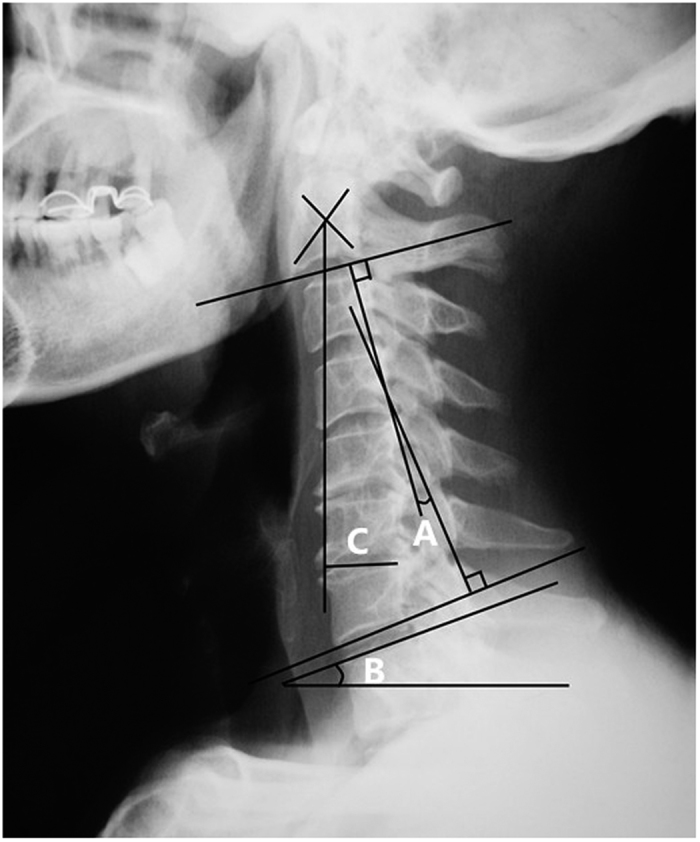
Radiographic measurements. (**A**) C2–7 Cobb angle. (**B**) T1 slope. (**C**) C2–7 SVA. SVA indicates sagittal vertical axis.

Anteroposterior and lateral X-ray images of the cervical spine obtained in the standing position were obtained at the preoperative stage and at a 1-year follow-up visit. Cervical lordosis was assessed by the C2–7 Cobb angle. The Cobb angle from C2–C7 was used as a measure of cervical alignment. The Cobb angle was defined as the angle formed by the inferior endplates of C2 and C7 on standing lateral radiographs. The C2–7 range of motion (ROM) was defined as the sum of the C2–7 Cobb angle during flexion and extension on lateral radiographs. The C2–7 sagittal vertical axis (SVA) was defined as the distance from the posterosuperior corner of C7 and the vertical line from the centre of the C2 body. The T1 slope or T1 sagittal angle was measured as the angle between a horizontal line and the superior endplate of T1 on a standing lateral radiograph^7^,^9^,^10^,^11^. The measurement methods are shown in Fig. 1. Postoperative kyphotic deformity was defined as a postoperative C2–7 angle less than −5 degrees after laminoplasty^6^.

**Figure 2:**
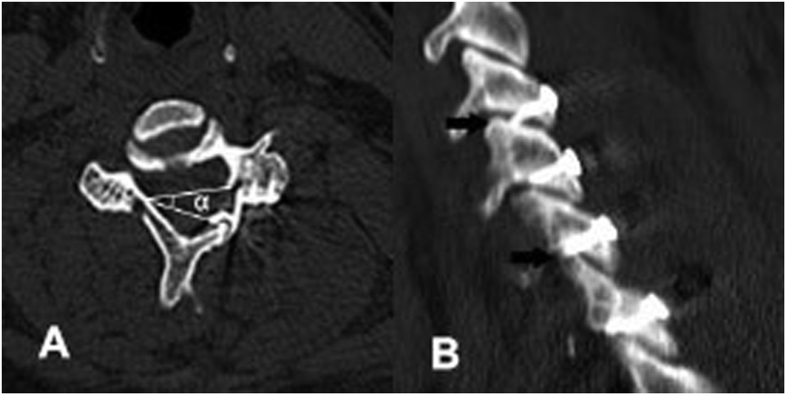
Radiographic measurements. (**A**) The angle α indicates the laminoplasty opening angle. (**B**) Facet joints were destroyed by the miniscrews (the black arrows).

CT scan data at 1 week postoperatively, including sagittal and axial sectional images, were used to identify the open angle and integrity of the facet joints. The opening angle of laminoplasty was defined as the average angle α value of opened laminae. The facet joint was recorded as destroyed when the articular surface of the facet joint was penetrated by the miniscrews that were used to fix the miniplate to the lateral mass (Fig. 2). Bony fusion of the hinge was evaluated using 6-month follow-up CT scan data. Bony fusion of the hinge could be identified only when the dorsal and ventral cortices of the ends of the hinge fracture were both fused^12^.

Preoperative and postoperative neurological function 1 year after surgery was assessed using the JOA score. The recovery rate of the JOA score was calculated using Hirabayashi’s method^13^ and was based on the following formula: recovery rate = (postoperative JOA score−preoperative JOA score/17−preoperative JOA score) × 100%. Comorbidities were also recorded, including cardiovascular, respiratory, endocrine, gastrointestinal, renal, psychiatric, rheumatological, and neurological diseases.

### Statistical analysis

Descriptive analysis for the patient population was conducted using means and standard deviations (SDs) for continuous variables and frequencies and percentages for categorical variables. Univariate analyses were performed to assess the association between factors and post-laminoplasty kyphotic deformity using the independent Student’s t-test for continuous variables and the chi-square or Fisher’s exact test for categorical variables. Variables with a P value less than 0.10 in univariate analysis was considered for multivariate analysis. Multivariate analysis was performed using multivariate logistic regression analysis. Adjusted odds ratios (ORs) with 95% confidence intervals (CIs) are presented with their respective P values. P < 0.05 was considered to represent a statistically significant difference. All analyses were performed using SPSS software (version 21.0; SPSS Inc., Chicago, IL).

## Results

### Clinical outcomes

Postoperative kyphotic deformity was observed at the 1-year postoperative follow-up in 21 (10.8%) patients. Patients who suffered from post-laminoplasty kyphosis were classified into the P group (n = 21), while those who did not comprised the NP group (n = 173).

The clinical data are shown in Table 1. There were 109 men and 85 women, ranging in age from 33 to 87 years, with a mean age of 62.9 years. The mean follow-up period was 31.5 months, ranging from 12 to 70 months. Laminoplasty was performed at C3–7 in 95 patients, at C3–6 in 34 patients, and at C4–7 in 65 patients. There was a significantly higher rate of patients in the P group (n = 19, 90.5%) who received cervical laminoplasty starting at the C3 level compared with those in the NP group (n = 110, 63.6%, P = 0.014). The preoperative JOA score was not significantly different between the two groups. However, the postoperative JOA score (P < 0.001) and recovery rate of the JOA score (P < 0.001) at the 1-year follow-up in the P group were inferior to those in the NP group. There were no significant differences in age, sex, body mass index (BMI), duration of myelopathy, smoking status, comorbidities, and duration of follow-up between the two groups.

### Radiographic outcomes

The radiographic data are shown in Table 2. The P group had a significantly greater C2–7 SVA (P = 0.003) and T1 slope (P < 0.001) than did the NP group. However, the C2–7 Cobb angle (P = 0.174), C2–7 ROM (P = 0.807), and open angle (P = 0.701) were not significantly different between the two groups. The mean number of facet joints that were destroyed in the P group was significantly higher than that in the NP group (P = 0.001). The hinge union rate at 6 months was not significantly different between the two groups (P = 0.501).

### Multivariate analysis

The CVLL, C2–7 SVA, T1 slope, and number of destroyed facet joints were finally included in multivariate logistic regression analysis. Multivariate analysis showed that the C2–7 SVA (OR = 1.085, P = 0.015), number of destroyed facet joints (OR = 1.132, P < 0.001), and CVLL at the C3 level (OR = 2.860, P = 0.021) were related to kyphosis after unilateral expansive open-door cervical laminoplasty in patients with CSM without preoperative kyphotic alignment (Table 3).

## Discussion

Unilateral expansive open-door cervical laminoplasty with miniplate fixation was first reported by O’Brien^14^ in 1996 and has recently become an increasingly popular method to treat multilevel CSM^15^,^16^. Although laminoplasty is the mainstay treatment option for CSM, it is associated with delayed postoperative problems, including kyphotic deformity^4^. Cervical kyphotic deformity is an important complication following laminoplasty that may be associated with pain and functional disability^17^. Cervical kyphotic deformity can prevent indirect decompression via a posterior cervical spinal cord shift and lead to postoperative residual anterior compression of the cervical spinal cord. We performed posterior decompression with laminoplasty only for patients without preoperative kyphotic alignment. However, interestingly, kyphotic deformity occurred at the 1-year postoperative follow-up in 21 (10.8%) of our patients.

Post-laminoplasty kyphotic deformity is likely multifactorial. In a retrospective cohort study, Michael *et al*.^8^ reported significantly less loss of cervical lordosis for plated open-door laminoplasty beginning at C4 compared with C3. Kim *et al*.^7^ showed that a high T1 slope preoperatively is a risk factor for postoperative kyphosis. The T1 slope is useful in evaluating sagittal balance, and it is most strongly correlated with the C2–7 SVA^18^. Recently, Sakai *et al*.^6^ showed that the centre of gravity of the head to the C7 SVA was a preoperative risk factor for kyphotic deformity after double-door laminoplasty for patients with CSM without preoperative cervical kyphotic alignment. However, a comprehensive study that included these and other underlying factors is lacking. In contrast to these previous studies, the present study, which used multivariate regression analysis, examined important factors that might be associated with kyphotic deformity after unilateral expansive open-door cervical laminoplasty. We found that the C2–7 SVA, the number of destroyed facet joints, and the CVLL were significantly associated with post-laminoplasty kyphosis.

Cervical sagittal balance based on the cervical SVA is an important determinant of clinical outcomes after cervical surgery. An increased C2–7 SVA due to head and neck tilting forward ultimately leads to more loss of lordosis. The T1 slope has constant morphological values within an individual and significantly influences sagittal balance of the cervical spine. Kim *et al*.^7^ reported that a high T1 slope was a predictive risk factor of kyphotic deformity after laminoplasty. In the present study, although the T1 slope showed a difference in univariate analysis, it was not an independent risk factor in multivariate analysis. A recent study reported that the T1 slope did not affect changes in sagittal alignment post-laminoplasty, which is similar to our result^19^. This finding indicates that theT1 slope might be a confounding factor for kyphotic deformity.

Our study also showed that the CVLL was another independent predictor of kyphotic deformity after unilateral expansive open-door cervical laminoplasty. The paraspinal muscles usually play an important role in developing kyphotic deformity after laminoplasty. However, there is no unified standard for assessing function of the paraspinal muscles. In our study, we selected CVLL to evaluate the effect of preservation of insertion of the deep extensor musculature to the C2 spinous process. Fujimura and Nishi^11^ analysed the cervical extensor musculature on CT images and plain radiographs that were obtained in 53 patients who had undergone laminoplasty. They reported a correlation between the deep nuchal muscle area and the curve index. Iizuka *et al*.^20^ previously used MRI to correlate operative repair of the semispinalis muscle with maintenance of lordosis following laminoplasty. They reported that the degree of repair of the deep extensor musculature affects postoperative cervical alignment. Furthermore, several studies have demonstrated the importance of C2 extensor muscle attachment in postoperative cervical spine alignment^20^,^21^. Iizuka *et al*.^22^ also showed that preservation of insertion of the deep extensor musculature to the C2 spinous process prevented significant loss of lordosis after laminoplasty. Anatomically, the distal portion of the C2 lamina is dorsal to, overlaps, and partially blocks the cephalad portion of the C3 lamina. Therefore, when laminoplasty is performed at the C3 level, some degree of muscle detachment of C2 is almost always unavoidable to obtain the exposure necessary for opening C3 on C2. In contrast, although laminoplasty starting at the C4 level requires at least some dissection of C3 muscular attachment, it can generally be performed without disturbing insertion of extensor muscle to C2.

Destroyed facet joints were also a novel factor related to kyphotic deformity after unilateral expansive open-door cervical laminoplasty. Although a miniplate can preserve the facet joint and prevent injury to the joint capsule on the hinge side, damage of the facet joint may unexpectedly occur on the open side. In the present study, we found that miniscrews that were used to fix the miniplate to the lateral mass might penetrate the facet joint surface and destroy the facet joint. Destroyed facet joints might affect stability of the cervical spine and induce nonbacterial inflammation, which may contribute to kyphotic deformity.

There are several limitations that need to be considered in our study. First, our study was retrospective in nature with a relatively small cohort size for evaluating such a rare incidence. Second, the period of the greatest extent of loss of lordosis after laminoplasty for CSM is unclear. Therefore, evaluation at 1 year postoperatively may not be sufficient. Third, the relationship between kyphotic deformity after laminoplasty and thoracolumbar or spino-pelvic parameters could not be confirmed because whole sagittal radiographs were not performed.

## Conclusions

The CVLL, C2–7 SVA, and destroyed facet joints might be associated with kyphosis after unilateral expansive open-door cervical laminoplasty in patients with CSM without preoperative kyphotic alignment.

## Additional Information

**How to cite this article**: Cao, J.M. *et al*. Multivariate analysis of factors associated with kyphotic deformity after laminoplasty in cervical spondylotic myelopathy patients without preoperative kyphotic alignment. *Sci. Rep.* **7**, 43443; doi: 10.1038/srep43443 (2017).

**Publisher's note:** Springer Nature remains neutral with regard to jurisdictional claims in published maps and institutional affiliations.

## Figures and Tables

**Table 1:** Clinical factors and univariate analysis of post-laminoplasty kyphotic deformity.

	Total	Group P	Group NP	P-value
Age (years)	62.9 ± 10.2	62.0 ± 8.7	63.1 ± 10.4	0.660
Sex (male: female)	109/85	15/6	94/79	0.136
BMI (Kg/ m^2^)	26.4 ± 7.1	27.9 ± 4.5	26.2 ± 7.3	0.129
Smoking (N/Total)	95/194	10/21	85/173	0.896
Comorbidities (N/Total)	93/194	8/21	85/173	0.339
Duration of myelopathy (mths)	18.8 ± 8.5	22.9 ± 16.0	18.4 ± 7.0	0.199
CVLL (N, %)				
C3	129(66.5%)	19(90.5%)	110(63.6%)	0.014
C4	65(33.5%)	2(9.5%)	63(36.4%)	
Follow-up (mths)	31.5 ± 14.5	32.0 ± 10.0	31.5 ± 15.1	0.827
Preoperative JOA score	8.8 ± 2.5	8.0 ± 2.2	9.1 ± 2.5	0.112
Postoperative JOA score	14.2 ± 2.8	11.4 ± 1.8	14.6 ± 2.7	<0.001
Recovery rate of JOA sore (%)	61.5 ± 14.5	31.9 ± 12.0	65.2 ± 10.7	<0.001

BMI: body mass index; CVLL: cephalad vertebral level undergoing laminoplasty; mths: months.

**Table 2:** Radiographic factors and univariate analysis of post-laminoplasty kyphotic deformity.

	Total	Group P	Group NP	P-value
C2–7 angle (degrees)	20.6 ± 10.1	17.7 ± 9.7	20.9 ± 10.1	0.174
C2–7 ROM (degrees)	39.2 ± 13.4	38.5 ± 12.4	39.3 ± 13.5	0.807
C2–7 SVA (mm)	20.4 ± 15.0	24.7 ± 10.1	19.1 ± 15.2	0.003
T1 slope (degrees)	20.0 ± 4.7	28.3 ± 3.0	18.9 ± 3.7	<0.001
Open angle (degrees)	35.6 ± 6.0	35.2 ± 7.0	35.7 ± 5.9	0.701
No. of facet joint destroyed	1.6 ± 1.4	2.9 ± 1.6	1.5 ± 1.2	0.001
Hinge union rate at 6 months (%)	93.8	90.5	94.2	0.501

ROM: range of motion; SVA: sagittal vertical axis.

**Table 3:** Multivariate analysis of factors associated with post-laminoplasty kyphotic deformity.

	OR	95% CI	P-value
T1 slope	1.016	0.677–1.120	0.158
C2–7 SVA	1.085	1.025–1.203	0.015
No. of facet joint destroyed	1.132	1.068–1.208	<0.001
CVLL at the C3 level	2.860	1.164–6.847	0.021

SVA: sagittal vertical axis; CVLL: cephalad vertebral level undergoing laminoplasty; OR: odds ratios; CI: confidence intervals.
